# Can luteal regression be reversed?

**DOI:** 10.1186/1477-7827-4-53

**Published:** 2006-10-30

**Authors:** Carlos M Telleria

**Affiliations:** 1 Division of Basic Biomedical Sciences, Sanford School of Medicine of The University of South Dakota, Vermillion, SD 57069, USA

## Abstract

The corpus luteum is an endocrine gland whose limited lifespan is hormonally programmed. This debate article summarizes findings of our research group that challenge the principle that the end of function of the corpus luteum or luteal regression, once triggered, cannot be reversed. Overturning luteal regression by pharmacological manipulations may be of critical significance in designing strategies to improve fertility efficacy.

## Background

The corpus luteum is a peculiar endocrine gland owing to its limited functional life. How long this gland lives and when it dies is dictated by a synchronized interplay of hormonally regulated events. It undergoes a complex process of formation or luteinization, which is followed by a period of active function that is mostly focused on the production of progesterone. Finally, the gland undergoes a process of regression associated with the decline in progesterone output and the demise of the tissue as a consequence of the programmed death of the luteal cells. A number of excellent review articles have been published in the last 6–7 years compiling the knowledge mastered on the molecular regulation of the formation, function and regression of the corpus luteum [1-13]. Yet, although luteal regression has been intensively studied, many of the regulatory mechanisms involved in loss of function and involution of the luteal structure are not completely understood. One fundamental question that remains without an answer is whether the process of luteal regression, once initiated, can be blocked or even reversed. In other words, can luteal function be rescued when it has already been impaired? Or instead, is luteal regression an irreversible event that cannot be modified when it progresses beyond a particular molecular step?

## Discussion

It is known that a cycling corpus luteum can survive longer if it is rescued by luteotropins. Physiologically this rescue takes place when the gland is fully functioning. For example in humans, chorionic gonadotropin produced by the trophoblast cells targets and rescues the corpus luteum at the mid-luteal phase of the menstrual cycle when the gland is at the maximal steroidogenic output attainable during non-pregnant cycles [8,14]. In the rat, vaginal stimulation during coitus triggers a neuroendocrine reflex that leads to the secretion of pituitary prolactin in a pattern of two daily surges. Prolactin then targets and rescues the corpus luteum in the morning of diestrus day 2 of the estrous cycle, when the capacity of the gland to produce progesterone is also maximal [15]. It is not known, however, whether the corpus luteum can be rescued while it is already in regression, and, consequently, not fully functional. Because luteal regression involves a complex and likely synchronized sequence of molecular events, it is rather difficult to determine when a pharmacological intervention could be made to rescue the corpus luteum making it fully functional again, or, in other words, "bringing it back to life."

In a model of luteal regression induced by the intraovarian administration of luteinizing hormone to day 19 pregnant rats, luteal regression was documented 24 hours later by the decreased activity of the luteal 3beta-hydroxysteroid dehydrogenase (3beta-HSD) enzyme (a marker of reduced steroidogenic output), the decreased intraluteal concentration of progesterone, and the reduced levels of progesterone in the circulation [16]. Forty eight hours after the treatment with luteinizing hormone the process of luteal regression was further highlighted by increased luteal activity of the enzyme 20alpha-hydroxysteroid dehydrogenase -20alpha-HSD, a marker of ongoing progesterone catabolism in rodents – and increased concentration of prostaglandin F2alpha (PGF2alpha) within the corpus luteum [16]. However, when progesterone was administered into the ovarian bursa 24 hours after the administration of luteinizing hormone -i.e. at a time when some of the signs of luteal regression had been already noticeable – the induction of luteal 20alpha-HSD activity was significantly blocked, serum progesterone concentration was significantly increased and intraluteal PGF2alpha concentration was significantly reduced [16]. In another example in which administration of the antiprogesterone mifepristone (popularly known as RU486) to day 18 pregnant rats resulted in premature parturition and signs of luteal regression, administration of androgens (androstenedione or testosterone) 24 hours later significantly prevented the declines in corpus luteum weight and serum progesterone concentration otherwise induced by the antigestagen [17]. Together all previous evidence suggest that even though luteal regression was occurring by the time the tropic hormonal support was given, the luteotropic hormones were still capable of deregulating some of the components of the luteal regression program.

Because androstenedione is the main circulating androgen in pregnant rats [18] and progesterone is the major steroid produced within the corpus luteum [6], one can speculate that these steroids may be responsible for protecting luteal cells from undergoing programmed death during most of the pregnancy. Consequently, as both progesterone and androstenedione decline at the time of parturition in this species, such lack of tropic hormonal support may trigger luteal regression. However, we have generated evidence that suggests that if a pharmacological intervention is made and androstenedione or progesterone becomes elevated at the proper time, luteal regression can be prevented. We have shown that increasing intraovarian levels of progesterone in late pregnant rats leads to the interference with PGF2alpha-induced 20alpha-HSD expression within the corpus luteum [19]. Moreover, by increasing the levels of circulating progesterone or androstenedione in rats after parturition (i.e. at a time when the luteal regression process is further more advanced when compared with that at the end of pregnancy), either pharmacologically by injecting the hormones [20,21], or physiologically by allowing the animals to lactate [22], apoptotic programmed death of the luteal cells could be significantly delayed as assessed by in situ 3' end DNA labeling and fragmentation of the DNA. This evidence suggests that the gland, even during regression, maintains a certain level of function in order to be rescued by an appropriate stimulus. In support of this hypothesis our recent data also show an increased expression of receptors for prolactin (main tropic hormone for the pregnant rat corpus luteum) and androgen in postpartum rat corpus luteum, after being temporally down-regulated at the end of pregnancy [20,22].

**Figure 1:**
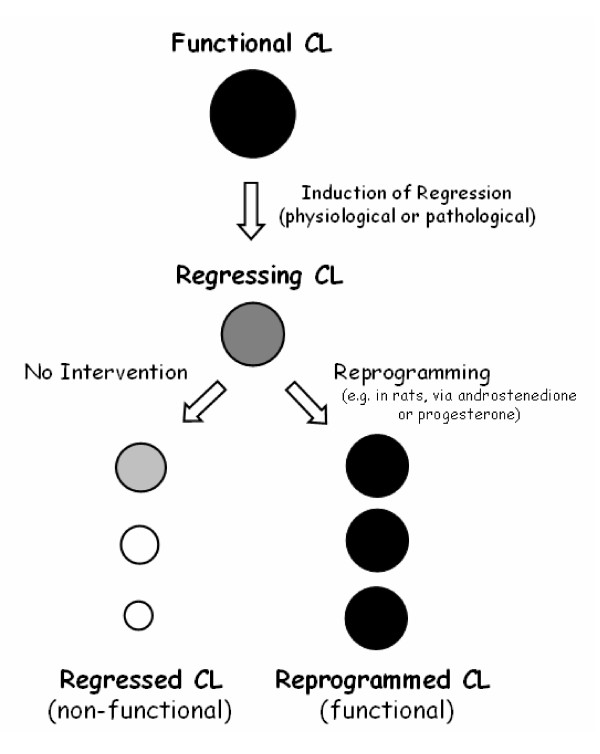
**Schematic representation of the functional reprogramming of regressing corpora lutea**. Support for this model comes from studies in rats, in which the corpus luteum (CL) can be rescued or "reprogrammed" while already regressing by the action of androstenedione or progesterone. Which factors/hormones/drugs can function as rescuers of regressing corpora lutea in other mammalian species, needs to be investigated.

Another example supporting the hypothesis that the corpus luteum maintains its capacity to be programmed back to its main role, even while regressing, came from the observation that there was always a larger response to androstenedione, in terms of progesterone producing capacity, from the luteal cells that were obtained from animals whose corpora lutea were not at their peaks of function in vivo. Thus, the addition of androstenedione to the culture media of luteal cells isolated from animals sacrificed on days 4, 9, 15, or 19 of pregnancy increased progesterone production over basal levels by 243, 39, 84 and 146%, respectively [23] – notice that in rats ovarian progesterone production peaks in between days 9 and 15 of pregnancy, on day 4 is relatively low yet raising, whereas on day 19 it is already declining [24]. We have also shown that androstenedione increased progesterone production by 99, 136, and 277% when added to cultured luteal cells obtained from animals sacrificed late in pregnancy on days 19, 20, and 21 respectively [17]. Again, these results clearly indicate that the maximal steroidogenic response to androstenedione was obtained from cells that were isolated from corpora lutea taken from day 21 pregnant rats; this is a time in pregnancy that is associated with the initiation of luteal regression as marked by the reduced progesterone output, when compared to that of days 20 and 19 of pregnancy. Altogether these studies suggest that although the luteal cells were apparently declining their progesterone output in vivo they still possess an intact progesterone-producing capacity that was manifested when they were taken away from the in vivo environment. From the analysis of these previous studies it is appealing to hypothesize that whereas the life of the corpus luteum appears to be "programmed" it may be able to be "reprogrammed" by a timely pharmacological intervention (Figure 1). This could be particularly helpful in humans where corpus luteum deficiency is usually circumvented by the administration of exogenous progesterone, a strategy that is not always successful in generating a secretory uterus to carry out a successful pregnancy until the ovarian/placental shift of progesterone production takes place [25-27]. By providing only progesterone, the potential effect of other factor/s being produced by the corpus luteum of early pregnancy that might contribute to implantation and early fetal development is discouraged. In this context of reasoning a better therapeutic strategy in patients with luteal dysfunction could be rescuing the full synthetic capacity of the corpus luteum of early pregnancy, rather than providing progesterone replacement. Consequently, based on the studies conducted in rodents, it seems that any pharmacological intervention capable of reprogramming this poorly functional corpus luteum associated with luteal dysfunction appears promising.

## Conclusion

Taken together, these previous studies conducted in rats and mostly generated using two luteotropic agents for this species, androstenedione and progesterone, strongly suggest that luteal regression can be interfered when it is already induced, by targeting at least two different steps in the process: i) rescuing the capacity of the gland to produce progesterone; and ii) interfering with programmed death of the luteal cells leading to a delay in the loss of luteal weight. When showing signs of poor efficiency in its steroidogenic output, if the corpus luteum could be hormonally reprogrammed to prolong its lifespan by preventing its demise, such achievement would be critical in designing new strategies to improve fertility efficacy.
